# 人工智能三维重建辅助规划胸腔镜肺段切除术的应用价值

**DOI:** 10.3779/j.issn.1009-3419.2023.102.28

**Published:** 2023-07-20

**Authors:** Zhizhong ZHENG, Meiyu REN, Bin LI, Jianbao YANG, Xiaoping WEI, Tieniu SONG, Yuqi MENG, Yuzhen CHEN, Qing LIU

**Affiliations:** 730030 兰州，兰州大学第二医院胸外科，兰州大学第二临床医学院; Department of Thoracic Surgery, Lanzhou University Second Hospital, Lanzhou University Second Clinical Medical College, Lanzhou 730030, China

**Keywords:** 人工智能, 三维重建, 磨玻璃结节, 肺段切除术, Artificial intelligence, Three-dimensional reconstruction, Ground-glass nodule, Segmentectomy

## Abstract

**背景与目的** 三维重建可辅助规划肺段切除手术，基于人工智能算法的三维重建软件正逐步应用于临床中。本研究旨在评估人工智能三维重建辅助规划胸腔镜肺段切除术的准确性及安全性。**方法** 共纳入兰州大学第二医院胸外科收治的90例经评估拟行胸腔镜肺段切除术的患者，术前使用人工智能三维重建软件制作肺三维图像并进行术前规划，术中保存手术录像，记录围手术期数据。选取38例患者的视频录像，探究人工智能三维重建对于手术规划的效能。将人工智能三维重建图像及Mimics 21软件重建结果与术中实际所见进行对比，比较两种重建方法对支气管、血管的检测及分型能力。**结果** 90例患者均在人工智能三维重建规划下完成手术，其中单一肺段切除57例（63.3%），联合亚段切除33例（36.7%）。人工智能三维重建对病变定位的准确率为100.0%，计算机断层扫描（computed tomography, CT）报告准确率为94.4%（85/90）。人工智能三维重建和Mimics 21软件的检测准确率分别为92.1%（35/38）和89.5%（34/38），解剖分型准确率分别为89.5%（34/38）和84.2%（32/38），总准确率分别为76.3%（29/38）和71.1%（27/38）。在对38例手术视频与重建图像的对比观察中发现，使用人工智能三维重建进行术前规划的靶段规划、手术入路、动脉离断、静脉离断、支气管离断的一致率分别为92.1%（35/38）、92.1%（35/38）、89.5%（34/38）、86.8%（33/38）、94.7%（36/38），总规划操作一致率为68.4%（26/38）。**结论** 使用人工智能三维重建辅助规划胸腔镜肺段切除术是准确且安全的。

## Abstract (en)

**Background and objective** The three-dimensional (3D) can assist in planning lung segmentectomy. 3D reconstruction software based on artificial intelligence algorithm is gradually applied in clinic. The aim of this study was to evaluate the accuracy and safety of 3D reconstruction assisted planning of thoracoscopic segmentectomy. **Methods** A total of 90 patients admitted to Department of Thoracic Surgery of Lanzhou University Second Hospital were evaluated for thoracoscopic segmentectomy. Before operation, artificial intelligence 3D reconstruction software was used to make 3D lung images and conduct preoperative planning. Surgical videos were saved during the operation and perioperative data were recorded. Video recordings of 38 patients were selected to explore the effectiveness of artificial intelligence 3D reconstruction for surgical planning. The results of artificial intelligence 3D reconstruction and Mimics 21 software reconstruction were compared with the actual results in the operation, and the detection and classification ability of bronchus and blood vessels of the two reconstruction methods were compared. **Results** All the 90 patients underwent artificial intelligence 3D reconstruction planning, including 57 patients (63.3%) with single lung segmentectomy and 33 patients (36.7%) with combined sub-segmentectomy. The accuracy of artificial intelligence 3D reconstruction for lesion localization was 100.0%, and the accuracy of computed tomography (CT) was 94.4% (85/90). The detection accuracy of artificial intelligence 3D reconstruction and Mimics 21 software was 92.1% (35/38) and 89.5% (34/38), and the anatomic typing accuracy was 89.5% (34/38) and 84.2% (32/38), and the total accuracy was 76.3% (29/38) and 71.1% (27/38). In the comparative observation of 38 surgical videos and reconstructed images, the consistent rates of target segment planning, surgical approach, artery dissection, vein dissection and bronchial dissection for preoperative planning using artificial intelligence 3D reconstruction were 92.1% (35/38), 92.1% (35/38), 89.5% (34/38), 86.8% (33/38) and 94.7% (36/38). The overall planning operational consistency rate was 68.4% (26/38). **Conclusion** It is accurate and safe to use artificial intelligence 3D reconstruction to assist planning thoracoscopic segmentectomy.

肺段切除术是肺切除手术中的常规术式，其最早由Churchill与Belsey^[1]^提出，主要用于治疗支气管扩张、结核等良性疾病。1973年，Jensik等^[2]^首次系统地报道了肺段切除术可能用于外科治疗早期肺癌。随着近期JCOG0802/WJOG4607L^[3]^及CALGB140503^[4]^两项著名临床研究的结果发布，肺段切除术对直径≤2 cm的肺癌的疗效得到了强有力的证实，因此这一术式在临床中也得到了进一步的广泛开展，肺段切除术已成为直径≤2 cm且影像学中包含磨玻璃成分的周围型肺癌可选择的治疗手段。

较肺叶切除术、肺楔形切除术等术式而言，肺段切除术需要识别和解剖更加精细的支气管及其伴行的血管并进行选择性离断，而肺段的解剖复杂多变，变异情况较多^[5,6]^，错误的离断可能会导致出血、术后咯血等严重并发症的发生。常规的胸部计算机断层扫描（computed tomography, CT）虽然可全面地提供患者肺部的影像信息，但仅依靠CT进行病变的定位，辨别气管、血管的变异，判断解剖及拓扑关系，并最终进行术前规划，对医生的阅片及空间想象能力有着很高的要求。基于CT的三维重建技术的出现，使得传统的二维影像被转化为直观的三维图像，从而实现病变、血管、支气管的三维可视化、直观展现解剖毗邻关系。Nakashima等^[7]^最早报道了肺三维重建用于规划并完成肺段切除（LS^6^），并在术前三维重建图像及术中均观察到了变异的A^6^c，证明了三维重建术前规划的重要意义。

近年来随着人工智能（artificial intelligence, AI）技术的发展和临床中对于成像更加准确且使用更加便利的三维重建软件的需求，基于AI的三维重建软件应运而生。本研究使用了基于AI的早期肺癌手术智能辅助决策系统（以下简称AI三维重建系统），该系统应用三维卷积神经网络在内的多种算法进行各种图像处理任务，除了常规报告结节的直径、密度、体积、平均CT值、恶性概率、早期肺腺癌的浸润亚型等数据外，可以全自动化地完成肺部三维图像制作。本中心的前期工作已经证实其在肺结节的检出及良恶性鉴别以及浸润亚型预测方面有一定的诊断价值^[8,9]^，本研究旨在探讨AI三维重建辅助规划胸腔镜肺段切除术的应用价值。

## 1 资料与方法

### 1.1 研究对象

本研究纳入兰州大学第二医院胸外科2022年2月1日至2023年4月30日收治的经评估拟行胸腔镜肺段切除术的患者，收集病例资料并录制手术视频。纳入标准：（1）直径≤2 cm的周围型肺结节，且在结节含有磨玻璃成分；（2）肺内多发病变需联合切除；（3）妥协性肺段切除术，即心肺功能较差的高龄患者或无法耐受肺叶切除的患者；（4）术后病理诊断为前驱腺体病变或肺癌。排除标准：（1）术前无完整影像资料者；（2）既往有肺部手术史；（3）拒绝接受本研究者。

研究共纳入90例患者，其中男性32例（35.6%），女性58例（64.4%），平均年龄（53.5±10.6）岁。肿瘤平均最大径（1.3±0.6）cm，肿瘤位于左侧40例（44.4%），右侧50例（55.6%）。本研究通过兰州大学第二医院伦理委员会审批（批准号：2023A-364）。

### 1.2 研究方法

#### 1.2.1 术前三维重建图像的制作及手术规划

将符合纳入、排除标准的患者胸部CT的DICOM图像上传至AI三维重建系统[点内（上海）生物科技有限公司]制作肺三维重建图像。由手术医生团队观察图像并制定手术方案及完成手术，术中录制手术视频。术前规划的内容主要包括：（1）观察病变的空间位置，用于术中病变定位；（2）根据病变大小、切缘范围确定需要切除的肺段；（3）规划手术入路（经叶裂、经前纵隔、经后纵隔）；（4）观察肺部动脉、静脉、支气管解剖形态及是否发生变异；（5）规划需选择性离断的动脉、静脉、支气管及离断、切除顺序。

术后选取38例患者，使用Mimics 21软件制作肺三维重建图像，并将Mimics 21重建图像、AI三维重建图像与手术视频实际所见进行对比，具体包括：（1）术中实际所见的动脉、静脉、支气管在三维重建图像上是否呈现并统计数量及准确率（图1，图2）；（2）对比两种方法的三维重建图像对血管、支气管的解剖分型是否与术中实际一致（图3）。

**图1:**
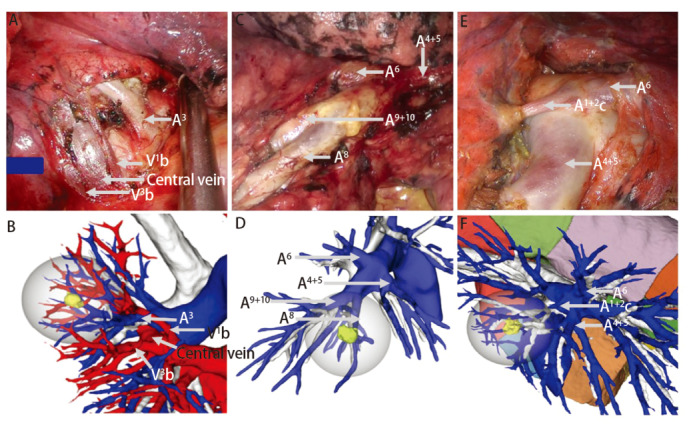
三维重建图像与术中探查一致。A、B：RS^1^切除的术中所见及AI三维重建图像；C、D：RS^8^切除的术中所见及AI三维重建图像；E、F： LS^1+2^切除的术中所见及AI三维重建图像。

**图2:**
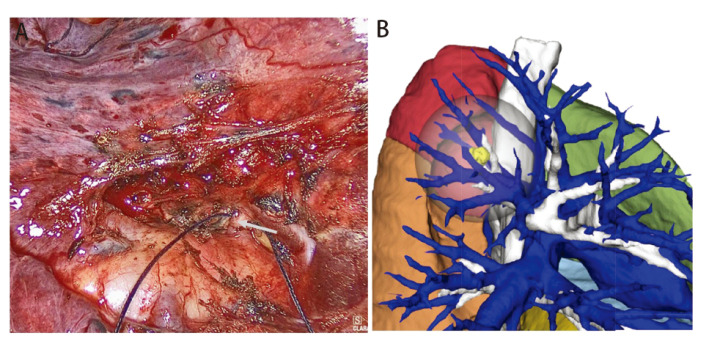
行RS^2^切除时术中探查到未被三维重建图像显示的动脉。A：术中探查到的Asc.A2；B：RS2切除的动脉、支气管三维重建图像。

**图3:**
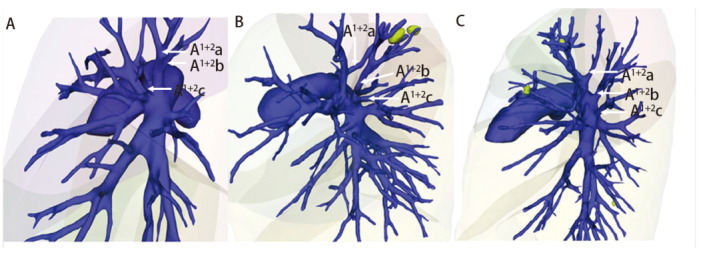
三种常见的LA^1+2^的解剖分型。A：A^1+2^a与A^1+2^b共干；B：A^1+2^a、A^1+2^b、A^1+2^c三支独立存在；C：A^1+2^a与A3共干。

#### 1.2.2 手术操作

（1）麻醉、体位：患者均采用静吸复合全身麻醉，双腔气管内插管并单侧肺通气。根据病变位置确定左或右侧卧位，手术野常规消毒铺巾。（2）手术切口及入路选择：手术均采用单孔或单操作孔胸腔镜操作，根据病变的不同位置选择皮肤切口。常规探查后根据三维图像的规划选择经叶裂、前纵隔或后纵隔入路。（3）AI三维重建图像辅助术中病变定位：术者依据三维重建图像显示的肺部病变所在的空间位置初步确定病变位置范围，术中可通过比较胸腔解剖标志、观察肺脏层胸膜表面、手指触摸或器械接触等进一步判断病变是否位于重建图像所显示的位置，并确定拟实际切除的肺段。（4）在三维重建图像引导下选择性动脉、静脉及支气管离断：从选定入路依照三维图像逐步分离解剖出需离断动脉、静脉、支气管并离断。（5）肺段间平面的确定及靶段切除：在完成选择性血管支气管离断后，使用改良“膨胀-萎陷法”显示肺段间平面，用直线型切割闭合器沿段间平面行肺段切除。取出肺组织后切开确定病变是否位于其中，判断是否切缘足够并送术中快速冰冻切片检查。（6）依据术中冰冻结果判断是否进一步行肺叶切除术，并行淋巴结采样或系统淋巴结清扫。常规行止血、水试验及胸腔引流管放置，清点纱布及器械无误后逐层缝合胸壁切口关胸。

#### 1.2.3 观察指标

（1）对于肺部病变位置的确定：记录根据三维重建规划而切除的具体肺段并明确病变是否位于其中，将此三维图像对于病变的定位结果与术前CT影像科医师报告进行对比。（2）对于肺支气管、血管的检测及分型：为了确定三维图像是否能够检测到手术中涉及的支气管、肺动脉、肺静脉并明确其解剖分型，由两名胸外科医生（其中一名为高级或副高级职称医师）在术后对手术视频及三维重建图像进行评估。当镜头识别的肺支气管、血管未在三维图像中显示时，将被记录为“未检测到”的支气管、血管。同时对视频显示的所能观察到的支气管、血管的解剖情况进行分型判断，并分别与两种方法得到的三维图像对比评估三维重建影像对于支气管、血管的解剖分型效能。（3）手术前规划效能：术前根据AI肺三维重建图像规划切除的肺段或亚段；拟定手术最佳入路，包括经前纵隔、经叶裂及经后纵隔入路；规划拟切断的动脉、静脉和支气管；规划依次切断结构的顺序。术中录制完整的手术视频，术后将手术视频与肺三维重建图像进行比较。（4）围手术期数据记录及随访：记录手术术式、切除肺段、术中是否中转肺叶切除、是否中转开胸以及病理结果。并对患者是否发生严重并发症、术后住院天数、是否出院后1个月再住院及是否出院后1个月死亡进行随访。

### 1.3 统计学分析

采用SPSS 25.0软件进行数据统计分析。将所有患者的人口统计学信息和基线特征制成表格并进行分析，正态分布的连续变量用均数±标准差（Mean±SD）表示，计数资料以例数和百分数（%）表示。

## 2 结果

### 2.1 一般资料

90例患者均在AI三维重建规划和引导下完成胸腔镜肺段切除术，患者的一般资料见表1。

**表1:** 患者临床资料（n=90）

Variable	Data
Age (Mean±SD, yr)	53.5±10.6
Gender	
Male	32 (35.6%)
Female	58 (64.4%)
Tumor location	
LS^1+2^	22 (24.4%)
LS^3^	4 (4.4%)
LS^4+5^	2 (2.2%)
LS^6^	3 (3.3%)
LS^8^	2 (2.2%)
LS^9^	2 (2.2%)
LS^10^	5 (5.5%)
RS^1^	13 (14.4%)
RS^2^	13 (14.4%)
RS^3^	6 (6.7%)
RS^6^	12 (13.3%)
RS^8^	4 (4.4%)
RS^9^	2 (2.2%)
Tumor size (Mean±SD, cm)	1.3±0.6
Type of surgery	
Segmentectomy	57 (63.3%)
Combined sub-segmentectomy	33 (36.7%)
Conversion from segmentectomy to lobectomy	4 (4.4%)
Conversion from VATS to thoracotomy	1 (1.1%)
Length of stay in hospital after surgery (Mean±SD, d)	4.1±1.8

VATS: video-assisted thoracic surgery.

### 2.2 肺部病变位置的确定

影像科医师根据CT报告对肿瘤位置的具体肺段的准确性为94.4%（85/90），依据AI肺三维图像切除肿瘤所在肺段后，所有病变均在其中，三维重建的准确率为100.0%（90/90）。

### 2.3 肺支气管、血管的检测及分型结果

将38例患者的手术视频与Mimics 21重建图像和AI重建图像对比后发现，96.4%的血管、支气管（292/303）被AI三维重建所识别，97.7%的血管、支气管（296/303）被Mimics 21所识别。具体识别的动脉、静脉、支气管数见表2。AI三维重建和Mimics 21重建对于38例患者的血管、支气管的解剖具体分型的准确性见表3。两种方法对支气管、血管的检测和解剖分型的效能见表4。

**表2:** AI三维重建对比Mimics 21重建的检测效能

Variable	No. of AVB involved in resection				Identification rate of AI			Identification rate of Mimics 21	
	AI	Mimics 21	Surgery		per-AVB basis	per-patient basis		per-AVB basis	per-patient basis
Artery	120	122	124		96.7%	94.7% (36/38)		98.4%	94.7% (36/38)
Vein	94	96	101		93.1%	92.1% (35/38)		95.0%	94.7% (36/38)
Bronchus	78	78	78		100.0%	100.0% (38/38)		100.0%	100.0% (38/38)
Overall	292	296	303		96.4%	92.1% (35/38)		97.7%	89.5% (34/38)

AVB: artery, vein and bronchus.

**表3:** AI三维重建对比Mimics 21重建的分型效能

Variable	Classification			Identification rate	
	AI	Mimics 21		AI	Mimics 21
Artery	34	36		89.5%	94.7%
Vein	33	35		86.8%	92.1%
Bronchus	38	38		100.0%	100.0%
Overall	32	34		84.2%	89.5%

**表4:** AI三维重建及Mimics21重建的规划准确性分析

Evaluation factor	Mimics 21	AI
Detection accuracy	89.5% (34/38)	92.1% (35/38)
Classification accuracy	84.2% (32/38)	89.5% (34/38)
Overall accuracy	71.1% (27/38)	76.3% (29/38)
Risky error rate	28.9% (11/38)	23.7% (9/38)

### 2.4 AI三维重建的术前规划效能

使用AI三维重建在术前完成患者的肺三维图像进行手术规划并术中引导，对切除靶段、手术入路、拟切断的动脉、静脉、支气管均可达到一定的规划效果，见表5，总术前规划与术中操作一致率为68.4%（26/38）。

**表5:** AI三维重建术前规划与术中操作的一致性分析

Index	Target segment planning	Surgical approach	Artery dissection	Vein dissection	Bronchial dissection	Overall
Coincident	35	35	34	33	36	26
Inconsistent	38	38	38	38	38	38
Accuracy rate	92.1%	92.1%	89.5%	86.8%	94.7%	68.4%

### 2.5 手术数据及随访

本研究中90例患者均顺利完成手术，其中行单纯肺段切除57例（63.3%），联合亚段切除33例（36.7%）。术中中转开胸1例（1.1%），中转切除肺叶4例（4.4%），平均术后住院天数（4.1±1.8）d，无术后严重并发症发生，无出院后1个月再住院病例，无出院后1个月死亡病例。

## 3 讨论

近年来，随着肺癌早期筛查的普及以及肺结节诊疗理念的不断发展，以肺结节为表现形式的早期肺癌检出率逐年增加。肺段切除术对于早期肺癌（限T1bN0）的治疗是安全且有效的，预示着肺段切除术或许在未来有望取代肺叶切除术成为部分早期肺癌的“金标准”手术治疗方式。而由于肺段切除术较肺叶切除、肺楔形切除难度更大，随着肺段切除术的广泛开展，临床中亟需一种方便快捷、有效安全的方法来辅助规划肺段切除术。我们所使用的AI三维重建系统在准确性方面取得了与传统三维重建软件相当的结果，同时操作简便、学习成本低，极大地省去了外科医生术前用于制作三维重建图像的时间，在减轻负担的同时提升了外科医生的工作效率。

在本研究中，我们观察了AI三维重建对比影像科医师对于肺部病变的定位效能，AI三维重建用于肺段切除术术前规划的效能，AI三维重建对比Mimics 21软件对于肺部支气管、血管的解剖识别及解剖分型效能，以及观察了使用此AI三维重建软件规划完成手术的短期安全性。我们在此将分别展开讨论。

早期肺癌的病灶体积较小且常需要进行肺楔形切除或肺段切除术治疗，在术中常难以仅通过肉眼观察或触摸等方法就能轻松定位。有研究^[10]^报道术中仅凭借手指触摸或者通过器械滑行定位病灶的成功率仅为30%。因此，术前辅助病变定位是十分必要的。目前临床中常用的术前辅助定位方法有：CT引导下经皮穿刺辅助定位技术，包括：经皮穿刺Hook-wire定位法^[11,12]^、经皮穿刺液体材料注射定位法等^[13]^、支气管镜下穿刺辅助定位技术（包括：电磁导航支气管镜下穿刺定位技术^[14]^）以及基于CT的三维重建辅助定位。这些方法各具优点同时也均有一定不足，目前CT引导下经皮穿刺辅助定位技术因其操作简单、手术时间短、成功率高、费用低廉，已成为临床最常用的术前定位方式。但其存在操作后气胸、出血、定位失败等风险及并发症，定位后患者应尽快进行手术。而电磁导航支气管镜下穿刺定位技术虽并发症较少并且更为安全，同时可以定位到解剖学上更难穿刺的部位，但因为其对于气管镜操作、麻醉的要求较高，同时费用高昂，在临床中难以推广。

在此背景下，基于CT的三维重建辅助定位法成为了准确性及安全性兼顾的一种定位方法。李响等^[15]^基于肺三维图像提出了病变距肺门深度百分比作为判定标准的概念。这一概念的提出，使得结节位置的描述更加客观，最终研究结果也同样认为基于深度比的肺结节三维位置定义方法较传统二维定义方法更为精确，有助于亚肺叶切除术术前规划。

在本研究中，我们对比了影像科医师基于胸部CT对病变所在肺段的诊断能力与三维重建方法的定位效能。通过对胸部CT图像的观察对肺部病变所在肺段进行判断并非一件困难的任务，绝大多数影像医师及胸外科医师均能通过病例资料得出准确结果。但在分析影像医师定位错误的5例病例后，我们发现对于远离段支气管的病变即外周性结节，尤其是位于两相邻肺段之间的病变，仅通过胸部CT去判断病变所在肺段仍是具有挑战的，而对于病变所在亚段的判断将会更难（图4）。得益于AI三维重建算法的稳定性，在计算机自动地识别出胸部CT中的病变后，可将病变准确无误地呈现在三维图像中，并且在完成该肺段手术切除后，90例患者的病变均位于三维图像呈现的肺段之中，准确率可达100.0%。

**图4:**
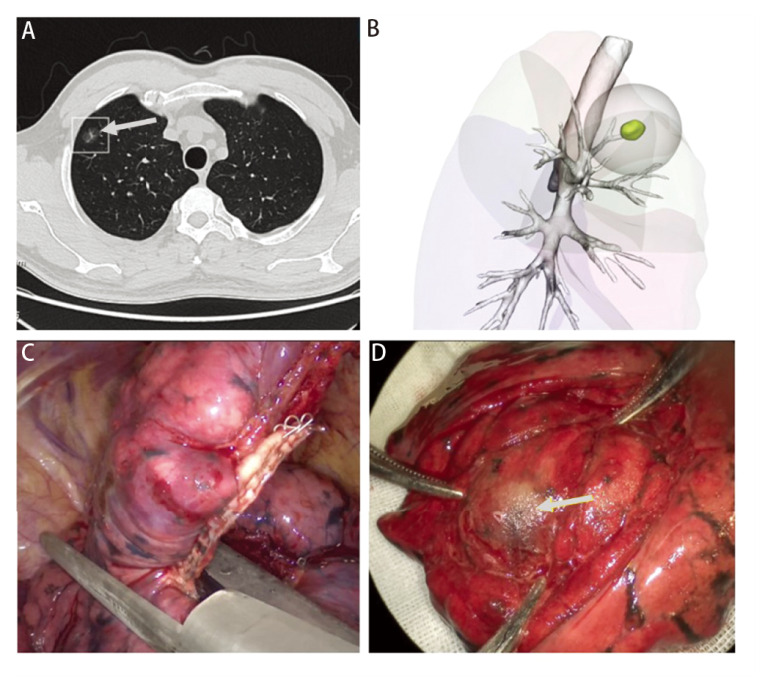
结节位于RS^1^与RS^3^之间，行RS^1^b+RS^3^切除后，结节位于切除肺组织中。A：右肺上叶磨玻璃结节1例；B：气管、结节、肺段的AI三维重建图像；C：手术切除RS^1^b+RS^3^；D：切除肺剖面可见病变。

除用于术前病变的定位外，多项研究及指南^[16,17]^均已提及三维重建可用于肺段切除术的术前规划，但少有文献研究术前三维重建规划对实际手术操作规划的具体效能，且目前尚无对于三维重建准确性和用于规划时一致性的客观评价标准。本研究中我们尝试提出了一些观察指标用于评估三维重建软件。我们发现，使用此三维重建图像进行术前规划可以很好地帮助术者明确操作过程，帮助术者在选择操作入路、拟定切除肺段及判断离断动脉、静脉、支气管，对减少手术中解剖结构的判断、避免过度解剖、最终缩短手术时间有着很大的帮助。

本研究中的总离断结构及顺序一致率为68.4%（26/38），分析那些未能按照术前规划完成手术的病例后，我们发现影响术中不能按照规划进行手术的主要原因常包括：（1）三维重建对于血管、支气管识别、解剖分型发生错误；（2）患者叶裂发育不良或胸腔内存在广泛黏连；（3）淋巴结较难分离导致离断血管、支气管时无法通过单一方向进行处理等。因此即使三维重建术前规划可以帮助术者准确选定最佳手术方案及精准判断需离断的结构，仍无法对术中的一些特殊情况（如黏连、叶裂发育、淋巴结情况）进行预判，需要术者依据术中探查情况进行手术决策。本研究所使用的AI软件暂无法直接提供手术规划方案，仍需手术医生观察三维图像并结合手术学相关知识进行手术方案的制定。随着本项研究的进一步深入及AI的发展，在未来或许可直接由AI制定肺段切除的手术方案，实现更加智能化的医疗。

为了评估AI三维重建软件的准确性，我们将术中所能观察到的解剖结构进行记录，并在AI三维重建和Mimics 21软件重建中观察是否正确呈现。Mimics 21和AI三维重建这两种方法的检测准确率和分型准确率分别可以达到89.5%、84.2%和92.1%、89.5%，总准确率为71.1%和76.3%，这与既往一些三维重建相关研究得出的结果^[18,19]^也是相近的。分析重建软件对动静脉数量重建结果不一致的原因主要在于重建目标的直径过小。既往的文献报道这些未能重建出的解剖结构直径绝大多数在2 mm以下。

为了评估AI三维重建辅助胸腔镜肺段切除的安全性，我们记录了所有患者的相关围手术期数据，包括：是否术中中转开胸、是否中转肺叶切除、术后住院时间、是否1个月内再住院以及是否1个月内死亡。在本研究的90例患者中，有1例患者因为术区淋巴结钙化且黏连致密导致动脉分离阻断困难，继续腔镜操作风险较大，遂中转开胸，最终完成LS^1+2^切除，术后分析血管、支气管解剖均与AI三维重建一致。另有4例患者在术中切除肺段后继续行肺叶切除术，其中1例剖开肺组织后见切缘不足2 cm，1例可见侵犯支气管，术后与三维重建图像对比可见解剖结构一致，但对于切缘与靶段规划存在判断失误，仅切除肺段不能满足R0切除要求。另2例因术中冰冻提示肿瘤侵犯脏层胸膜而继续行肺叶切除。

本研究未具体对比两种重建方法制作三维图像具体所需时间，但相较于传统三维重建软件，AI三维重建软件的操作简便，功能更加丰富。仅需要将患者的胸部CT的DICOM文件导入AI系统中即可得到肺三维重建图像，不再需要既往重建软件人工进行病变、支气管、血管、肺叶肺段分界、安全切缘等逐个重建，省去了动脉、静脉的识别区分等复杂过程，极大地减少了医生对于传统重建软件的学习成本和制图过程的时间成本。而本研究所使用的AI三维重建以及Mimics 21软件三维重建软件均只用于科学研究，暂未正式投入临床使用，因此未增加患者诊疗期间的额外费用。在AI三维重建正式应用于临床诊疗后，我们将对AI重建对患者诊疗费用方面的影响展开进一步讨论。

综上所述，本研究所使用的AI三维重建系统对于胸腔镜肺段切除术的术前规划和术中辅助引导具有很大帮助，较Mimics 21软件拥有相当的准确性，同时可极大程度地省去外科医生用于制图的时间，在未来的数字化、精准化、个体化医疗中，有着很大的推广价值。本研究存在一定的局限，如未设置对照组，进而观察术中时间、出血及并发症发生情况，也并未对所有患者的远期疗效、远期复发和生存情况进行随访，今后还需开展更深入的研究进一步探索。

**Competing interests**

The authors declare that they have no competing interests.

**Author contributions**

Zheng ZZ, Ren MY and Li B conceived and designed the study. Zheng ZZ performed the experiments. Yang JB, Wei XP, Song TN, Meng YQ participated in the completion of the operations. Zheng ZZ, Chen YZ, Liu Q analyzed the data. All the authors had access to the data. All authors read and approved the final manuscript as submitted.

## References

[1] Churchill ED, Belsey R. Segmental pneumonectomy in bronchiectasis: the lingula segment of the left upper lobe. Ann Surg, 1939, 109(4): 481-499. doi: 10.1097/00000658-193904000-00001 17857340 PMC1391296

[2] Jensik RJ, Faber LP, Milloy FJ, et al. Segmental resection for lung cancer. A fifteen-year experience. J Thorac Cardiovasc Surg, 1973, 66(4): 563-572. 4356889

[3] Saji H, Okada M, Tsuboi M, et al. Segmentectomy versus lobectomy in small-sized peripheral non-small-cell lung cancer (JCOG0802/WJOG4607L): a multicentre, open-label, phase 3, randomised, controlled, non-inferiority trial. Lancet, 2022, 399(10335): 1607-1617. doi: 10.1016/S0140-6736(21)02333-3 35461558

[4] Altorki N, Wang X, Kozono D, et al. Lobar or sublobar resection for peripheral stage IA non-small-cell lung cancer. N Engl J Med, 2023, 388(6): 489-498. doi: 10.1056/NEJMoa2212083 36780674 PMC10036605

[5] Ferry Jr RM, Boyden EA. Variations in the bronchovascular patterns of the right lower lobe of fifty lungs. J Thorac Surg, 1951, 22(2): 188-201. doi: 10.1016/s0096-5588(20)31267-8 14861933

[6] Wang Z, Sun Y, Zhang Q, et al. Uniportal VATS right superior lobectomy: management of pulmonary vein variation: a case report. J Cardiothorac Surg, 2020, 15(1): 45. doi: 10.1186/s13019-020-1088-3 32103769 PMC7045594

[7] Nakashima S, Watanabe A, Ogura K, et al. Advantages of preoperative three-dimensional contrast-enhanced computed tomography for anomalous pulmonary artery in video-assisted thoracoscopic segmentectomy. Eur J Cardiothorac Surg, 2010, 38(3): 388. doi: 10.1016/j.ejcts.2010.02.037 20362459

[8] Yin C, Mao WJ, Li B, et al. Study on the application of artif icial intelligence system in the detection and differentiation of benign and malignant pulmonary nodules. Zhonghua Xiongxinxueguan Waike Zazhi, 2020, 36(9): 553-556.

[9] Su ZP, Mao WJ, Li B, et al. Clinical study of artificial intelligence-assisted diagnosis system in predicting the invasive subtypes of early-stage lung adenocarcinoma appearing as pulmonary nodules. Zhongguo Feiai Zazhi, 2022, 25(4): 245-252. 35477188 10.3779/j.issn.1009-3419.2022.102.12 PMC9051300

[10] Ciriaco P, Negri G, Puglisi A, et al. Video-assisted thoracoscopic surgery for pulmonary nodules: rationale for preoperative computed tomography-guided hookwire localization. Eur J Cardiothorac Surg, 2004, 25(3): 429-433. doi: 10.1016/j.ejcts.2003.11.036 15019673

[11] Chen S, Zhou J, Zhang J, et al. Video-assisted thoracoscopic solitary pulmonary nodule resection after CT-guided hookwire localization: 43 cases report and literature review. Surg Endosc, 2011, 25(6): 1723-1729. doi: 10.1007/s00464-010-1502-3 21181200

[12] Lv XY, Yang YH, Hu J, et al. Clinical application of CT-guided preoperative pulmonary nodule localization technique. Zhongguo Feiai Zazhi, 2011, 14(5): 418-420. 21569647 10.3779/j.issn.1009-3419.2011.05.07 PMC6000330

[13] Shao F, Yang RS, Zou W, et al. The application of preoperative CT-guided puncturing and methylxanthine chloride staining in treatment of the small pulmonary nodules with video assistant thoracic surgery. Linchuang Feike Zazhi, 2012, 17(10): 1840-1841.

[14] Luo K, Lin Y, Lin X, et al. Localization of peripheral pulmonary lesions to aid surgical resection: a novel approach for electromagnetic navigation bronchoscopic dye marking. Eur J Cardiothorac Surg, 2017, 52(3): 516-521. doi: 10.1093/ejcts/ezx114 28459951

[15] Li X, Zhu YN, Wu WB, et al. Identification of pulmonary nodule location in three dimensional images and its clinical significance. Zhongguo Xiongxinxueguan Waike Linchuang Zazhi, 2021, 28(3): 305-310.

[16] Thoracic Surgery Branch of China International Exchange and Promotive Association for Medical and Health Care, Lung Cancer Prevention Branch of China International Exchange and Promotive Association for Medical and Health Care, Thoracic Surgery Specialty Committee of Health Exchanges and Cooperation Association, et al. Chinese expert consensus on the application of integrated 3D reconstruction with artificial intelligence in thoracic surgery across the Taiwan straits. Zhongguo Xiongxinxueguan Waike Linchuang Zazhi, 2023, 30(5): 641-646.

[17] Thoracic Surgery Committee, Department of Simulated Medicine, Wu Jieping Medical Foundation. Chinese Experts Consensus on artificial intelligence assisted management for pulmonary nodule (2022 version). Zhongguo Feiai Zazhi, 2022, 25(4): 219-225. 35340198 10.3779/j.issn.1009-3419.2022.102.08 PMC9051301

[18] Hagiwara M, Shimada Y, Kato Y, et al. High-quality 3-dimensional image simulation for pulmonary lobectomy and segmentectomy: results of preoperative assessment of pulmonary vessels and short-term surgical outcomes in consecutive patients undergoing video-assisted thoracic surgery. Eur J Cardiothorac Surg, 2014, 46(6): e120-e126. doi: 10.1093/ejcts/ezu375 25342848

[19] Chen X, Wang Z, Qi Q, et al. A fully automated noncontrast CT 3‐D reconstruction algorithm enabled accurate anatomical demonstration for lung segmentectomy. Thorac Cancer, 2022, 13(6): 795-803. doi: 10.1111/1759-7714.14322 35142044 PMC8930461

